# Molecular and comparative genomic analyses reveal evolutionarily conserved and unique features of the *Schizosaccharomyces japonicus* mycelial growth and the underlying genomic changes

**DOI:** 10.1007/s00294-021-01206-y

**Published:** 2021-08-24

**Authors:** László Attila Papp, Lajos Ács-Szabó, Gyula Batta, Ida Miklós

**Affiliations:** Department of Genetics and Applied Microbiology, Faculty of Science and Technology, University of Debrecen, Egyetem tér 1, Debrecen, 4032 Hungary

**Keywords:** *Schizosaccharomyces japonicus*, Dimorphism, Hyphae, Mycelial growth, Transcriptional profiling, Bioinformatic analysis

## Abstract

Fungal pathogens, from phytopathogenic fungus to human pathogens, are able to alternate between the yeast-like form and filamentous forms. This morphological transition (dimorphism) is in close connection with their pathogenic lifestyles and with their responses to changing environmental conditions. The mechanisms governing these morphogenetic conversions are still not fully understood. Therefore, we studied the filamentous growth of the less-known, non-pathogenic dimorphic fission yeast, *S. japonicus*, which belongs to an ancient and early evolved branch of the Ascomycota. Its RNA sequencing revealed that several hundred genes were up- or down-regulated in the hyphae compared to the yeast-phase cells. These genes belonged to different GO categories, confirming that mycelial growth is a rather complex process. The genes of transport- and metabolic processes appeared especially in high numbers among them. High expression of genes involved in glycolysis and ethanol production was found in the hyphae, while other results pointed to the regulatory role of the protein kinase A (PKA) pathway. The homologues of 49 *S. japonicus* filament-associated genes were found by sequence alignments also in seven distantly related dimorphic and filamentous species. The comparative genomic analyses between *S. japonicus* and the closely related but non-dimorphic *S. pombe* shed some light on the differences in their genomes. All these data can contribute to a better understanding of hyphal growth and those genomic rearrangements that underlie it.

**Supplementary Information:**

The online version contains supplementary material available at 10.1007/s00294-021-01206-y.

## Introduction

Many microorganisms are able to grow over a wide range of environmental factors because they have sophisticated cell processes, which ensure their cell proliferation and survival. One of these cell processes is dimorphism, which means that cells can form unicellular yeast cells or mycelia depending on nutrient supply or other environmental factors (reviewed in Biswas et al. 2007 and Whiteway and Bachewich 2007). Switching from yeast to hypha-phase means that cells become elongated, grow in a unipolar fashion and produce chains of cells (pseudohyphae) or long true invasive hyphae (Sipiczki et al. 1998a, b; reviewed in Ernst 2000 and Sudbery et al. 2004). Several external factors, such as low glucose concentration, changes in pH or temperature, presence of serum, peptone or certain stress factors can stimulate mycelial growth, which can contribute also to the virulence of these microorganisms (Buffo et al. 1984; Dede and Okungbowa 2009, reviewed in Biswas et al. 2007 or Ernst 2000; Szabo 1999, or Ceccato-Antonini and Sudbery 2004; Alby and Bennet 2009; Lo et al. 1997; Kumamoto and Vinces 2005; Brand 2012).

Previous studies have revealed that one of the fission yeasts, the *Schizosaccharomyces japonicus* belongs to dimorphic microorganisms (Sipiczki et al. 1998a, b). Its mycelial growth was regulated by the nutritional gradient and depended on alteration of the cAMP level, pH and temperature (Sipiczki et al.1998a, b; Sipiczki et al. 1998a; Papp et al. 2014). The presence of certain amino acids, light or DNA damage could also influence the length of its hyphae (Papp et al. 2014; Okamoto et al. 2013; Furuya and Niki 2010). Although this species is distantly related to budding yeasts, its hyphal growth could be induced by FBS (Fetal Bovin Serum), similarly to *Candida albicans* or *Yarrowia lipolytica* (Papp et al. 2014; Mackenzie 1962; Joshi et al. 1973; Perez-Campo and Dominguez 2001; Kim et al. 2000). At the same time, remarkable differences can also be found between the mycelia of fission and budding yeasts. *Candida albicans* hyphae have Spitzenkörper, while the *S. japonicus* does not (Crampin et al. 2005; Kinnaer et al. 2019). *S. japonicus* mycelial growth increased at pH 4–7, while pH 4 inhibited the hyphae formation of *Candida* (Papp et al. 2014; Konno et al. 2006). Hyphae of the most filamentous fungi are multinuclear, while *S. japonicus* hyphae remain mononuclear (Sipiczki et al. 1998a; Kinnaer et al. 2019).

Because of the similarities and differences mentioned above and because the fission yeasts have several ancient features (they share important cell processes with metazoans and have remarkably conserved gene content) (Sipiczki 2000; Rhind et al. 2011; Kuramae et al. 2006), the aim of this study was to further investigate *S. japonicus’*s mycelial growth, to reveal its filament-associated genes, as well as its evolutionarily conserved and species-specific features.

Our study revealed the *S. japonicus’*s genes involved in hyphal growth. We determined the gene functions and GO categories, which showed the complexity of this process. Our data suggested strong glycolysis and ethanol production in the hyphae and revealed the regulatory role of the PKA pathway. Comparison of the *S. japonicus* transcriptional data with other dimorphic and filamentous species revealed the conserved genes of hyphae formation, while the comparative genomic analysis of *S. japonicus* and the closely related but non-dimorphic *S. pombe* shed some light on those genetic changes which could have contributed to the different dimorphic capacities of the two species.

## Materials and methods

### Strains

The *S. japonicus var. japonicus* wild-type yeast strain (7–1) (CCY-44-5-1, CBS354, ATCC 10660) and *S. pombe* (2-1210) *ura4-D18* h^90^ were used in this study.

### Media

The YEA (2% D-glucose, 1% yeast extract (Scharlau), 2% agar) and YPA (2% glucose, 1% yeast extract (Scharlau), 1% (*w*/*v*) casein tryptone (Scharlau), 2% agar) were used as a standard culture media. The Petri dishes were incubated at 30 °C.

YPG (YEA solidified with 10% gelatin instead of agar) was used for the culturing of the yeast-phase and hyphal-phase cells necessary for RNA isolation (Papp et al. 2021).

The transformant *S. pombe* cells were spread onto EMMA + 15 µM thiamin, while the morphology of the transformant cells was investigated on EMMA (Mitchison 1970).

### Effect of isoamyl-alcohol, higher glucose concentration or presence of iron on the mycelial growth

YEA medium and YEA supplemented with 5% glucose, or 200 µM FeCl_3_, or 0.25% or 0.5% isoamyl-alcohol were prepared. The *S. japonicus* cells were streaked onto the surface of these agar plates and incubated at 30 °C. Length of the hyphae was photographed and measured after 5 days. The results were also checked after 12 days.

### Total RNA isolation

RNA was extracted from yeast-phase cells and hyphae. The culture conditions and modifications of the RNA isolation protocol (Lyne et al. 2003) can be found in the previous article (Papp et al. 2021). The most important modifications were the application of glass beads to achieve stronger break of the mycelial wall and RNA was extracted from cytoplasm-filled tips of the true invasive hyphae (instead of whole mycelia) which were grown on gelatin solidified culture medium (step-by-step protocol is in Papp et al. 2021).

### RNA sequencing strategy

To obtain global transcriptome data, the high throughput mRNA sequencing analysis was performed on Illumina sequencing platform. Total RNA sample quality was checked on Agilent BioAnalyzer using Eukaryotic Total RNA Nano Kit according to the manufacturer’s protocol. Samples with RNA integrity number (RIN) value 7 were accepted for the library preparation process.

Raw reads were aligned to the reference genome (*Schizosaccharomyces japonicus* yFS275). Tophat and Cufflinks bioinformatics tools were used for mapping and generating expression values.

The library preparations and the sequencing run were performed by UD-GenoMed Kft. and the Genomic Medicine and Bioinformatics Core Facility of Department of Biochemistry and Molecular Biology, Faculty of Medicine, University of Debrecen, Hungary. The data came from three separate experiments.

### RT-PCR analysis

To validate the RNA sequencing data, RT-PCR was performed with a few intron-containing and randomly selected genes. Two genes were up-regulated (SJAG_03283, *crp79* polyA binding protein and SJAG_04575, *meu6* meiotic chromosome segregation protein) and one gene was down-regulated (SJAG_05398, COX assembly mitochondrial protein) (Table S1).

Total RNA was extracted from yeast-phase cells and hyphae as described previously (Papp et al. 2021). cDNA synthesis was performed with the Thermo Scientific Revert Aid First Strand cDNA Synthesis Kit (K1621).

For the RT-PCR (Bio-Rad IQ5 real-time PCR system) reaction, SsoAdvanced Universal SYBR® Green Supermix (Bio-Rad, 1725272) reagent was used with final primer concentration of 0.2 mM. The primers are listed in Table 1. Serial dilutions of cDNA (1/5, 1/25, 1/125, 1/525) were prepared to generate standard curves for each reaction. All PCR reactions were performed in triplicates. PCR conditions were as follows: 98 °C for 2 min, 40–50 cycles: 98 °C 5 s, 57 °C 20 s. Melt curve was also generated according to the company instructions. The experiments were repeated at least twice using cDNA from different biological repeats. Data were analyzed with the software (Bio-Rad iQ5 2.0) supplied with the qPCR instrument, expression levels were normalized to *sce3*^+^ transcription data and outlying data were removed during analysis.

**Table 1:** Primers used in this study

Collection number and name of the primer	Sequence (5′–3′)	Position of the primers
1291 Crp79F	TTCTCTCAATACGGAAACG	exon1–exon2 border
1292 Crp79R	GGCTTTGACTGTAATTTTGC	exon3–exon2 border
1293 MEU6F	CGGCATTCTTCCTCATTC	exon1–exon2 border
1294 MEU6R	GACTCGGTCGCTGTTTTATC	exon2
1299 SJAG_05398R	TCGAGAATCACGTAACGATAC	exon2
1300 SJAG_05398F	CTTGATGGAAGATCGGAAAG	exon1–exon2 border
197 sce3F	GTCCGAGGGTGAGATTACCA	exon4
198 sce3R	GAACTCAACGTAGGCGAAGC	exon4
1310 Nrg1 Rev	GCTCGGATCCTTAGGAGGACAATAGGGATG	*nrg1* gene
1311 Nrg1 Forw	TGGTGGTGGTGGTTCTGGTGGTGGTGGTTCTGGTGGTGGTGGTTCTATGAGCGCCTCATTGTGTGT	*nrg1* gene
588 pREP F	GTCATTCGGCAATGTGCAGC	nmt1 promoter of the pREP42 vector

## Bioinformatics

### Source of the protein sequences

Protein sequences were gained from the Uniprot and the Pombase databases (https://www.uniprot.org/uniprot, http://www.pombase.org).

### Identification of orthologous proteins

Orthologous proteins were identified by reciprocal BLASTp analysis performed on the website of NCBI (http://blast.ncbi.nlm.nih.gov). When the reciprocal BLASTp analyses did not give a clear result, the homologous *S. pombe* genes were also searched using phmmer program (https://www.ebi.ac.uk/Tools/hmmer/search/phmmer) and synteny analyses (Ács-Szabó et al. 2018).

### Identification of GO terms

GO numbers and categories were obtained from the *S. pombe* database (http://www.pombase.org) (Lock et al. 2018) using *S. pombe* homologous genes.

### Identification of evolutionarily conserved genes of hyphal growth

Reciprocal BLASTp analyses were carried out with the *S. japonicus* protein sequences in https://fungi.ensembl.org. To find the *Taphrina deformans* homologous sequences we used the https://mycocosm.jgi.doe.gov database. RNA sequencing data of *C. albicans* and *H. capsulatum* were obtained from https://www.ncbi.nlm.nih.gov/geo/query/acc.cgi?-acc=GSE19583 based on Epp et al. (2010) and Gilmore et al. (2015).

### Source of the evolutionary rates

Data set of evolutionary rates of the fission yeasts protein sequences was obtained from Rhind et al. (2011).

### Investigation of chromosomal localization

Chromosomal localization coordinates of the fission yeast genes were obtained from Pombase and from the fungal ftp server of the Broad Institute (ftp://ftp.broadinstitute.org/pub/annotation/fungi/schizosaccharomyces/). Synteny analyses of certain genes between *S. japonicus* and *S. pombe* were performed manually using orthology inference and the genomic coordinates of the concerning genes. Synteny information on *S. octosporus* and *S. cryophilus* was obtained from Ács-Szabó et al. (2018).

Small-scale collinearity and gene losses were depicted with the online tool SimpleSynteny (https://www.dveltri.com/simplesynteny/) (Veltri et al. 2016). Localization of the *S. japonicus* genes with altered expression levels was displayed by using the OrthoClusterDB online platform (http://genome.sfu.ca/cgi-bin/orthoclusterdb/runortho.cgi) (Ng et al. 2009).

### Phylogenetic analysis

Phylogenetic tree was created at the website of Phylogeny.fr (http://www.phylogeny.fr/) (Dereeper et al. 2008) using certain protein sequences of high copy number genes of *S. japonicus*. The sequences were submitted to a manually adjusted workflow consisting of MUSCLE for alignment, GBLOCKS for the curation of the alignment and PhyML with WAG substitution model for phylogeny. The number of substitution rate category was adjusted to 4, gamma distribution parameter and proportions of invariable sites were both estimated. Branch support was estimated with an approximate likelihood-ratio test (aLRT) (Anisimova and Gascuel 2006). The tree was displayed with FigTree v1.4.4 (http://tree.bio.ed.ac.uk/software/figtree/). The protein sequence of SJAG_04799 was used as outgroup.

### Statistical analyses

Normal distributions of the data were tested by Shapiro–Wilk and Anderson–Darling tests. Since most of our datasets proved not to be normally distributed, Mann–Whitney *U* test was performed in the case of pairwise scenarios. Kruskal–Wallis test was used for multiple comparison, followed by pairwise Dunn test as post hoc test with Bonferroni corrections. *P* values were considered significant below the alpha level 0.05. All statistical analyses were performed in PAST v.3.20 software (https://folk.uio.no/ohammer/past/) (Hammer et al. 2001) and in Microsoft Office Excel 2016.

### Cloning of the *S. japonicus nrg1* gene

The *nrg1* gene was amplified from the *S. japonicus* genomic DNA with the 1310–1311 primers (Table 1) and the following parameters: 98 °C 1 min, 98 °C 30 s, 58 °C 30 s, 72 °C 30 s (30X), 72 °C 10 min. The PCR fragment (amplified with the NEB Phusion high-fidelity DNA polymerase) was inserted into the NdeI site of the pREP42 (Maundrell 1993). This vector has an inducible *nmt1*^+^ promoter which is regulated by thiamine. Orientation of the gene was checked by PCR (primers 588–1310). Morphology and size of the transformant cells cultured on minimal medium EMMA (*nmt1*^+^ promoter induced) (30 °C after 1 days) were investigated under an Olympus BX40 microscope. To calculate the ratio of the longer cells, 300 cells were investigated in both transformant cell populations. Localisation of the Nrg1 protein was checked by Olympus BX40 fluorescent microscopy.

### Transformation of the *S. pombe* cells

The uracil auxotrophic cells (2-1210) were transformed by the electroporator method (Gene Pulser Xcell-BioRad), according to the manufacturer’s protocol.

## Results

### Identification of the genes involved in *S. japonicus* mycelial growth

**Fig. 1:**
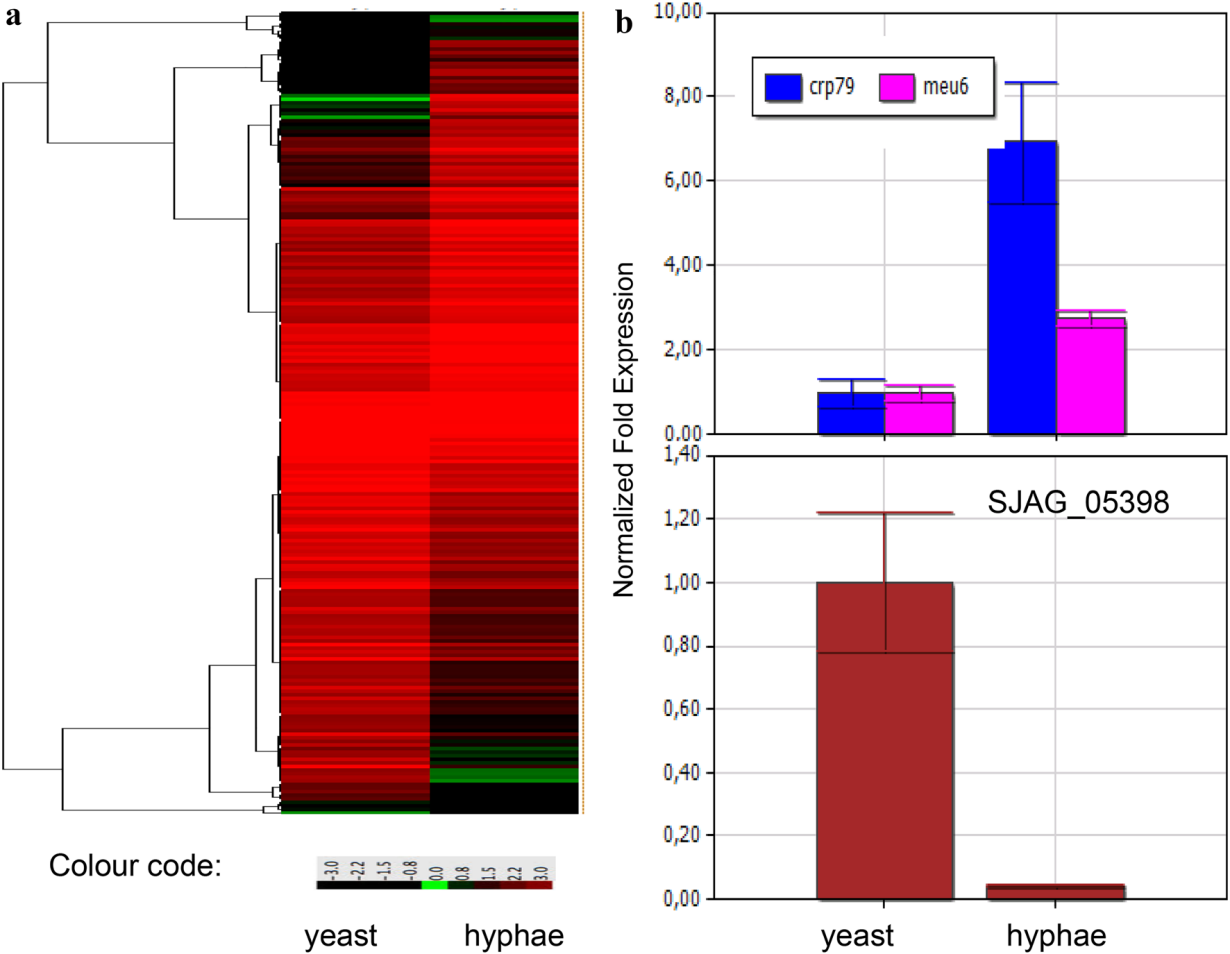
Heat map of the gene expression data and RT-PCR validation. Heat map was created from RNA sequencing data and showed differentially expressed genes in the hyphae, compared to the yeast-phase cells (**a**). The data were obtained from three separate experiments. Normalised expression of *crp79*, *meu6* and SJAG_05398 genes obtained with RT-PCR analyses (**b**)

To identify the genes involved in mycelial growth of the fission yeast *S. japonicus*, we performed RNA sequencing analysis. It revealed that the expression of more than 2000 genes changed in a statistically significant manner. Later we selected and investigated those genes whose log_2_ fold changes were at least ± 1.5 or higher (Fig. 1a). 191 genes were up-regulated, while 212 genes were down-regulated, compared to the yeast-phase cells. Their functions and GO categories were determined based on the homologous proteins of the closely related *S. pombe* (Table S1) (Lock et al. 2018). Interestingly, the filament-associated genes belonged to very different GO categories (Table S1, Table 2). Genes of transport and metabolic processes appeared especially in high numbers among them. The genes of vesicle-mediated transport or the catabolic processes were mostly up-regulated, while the genes of mRNA-, tRNA metabolic processes or DNA replication were mostly down-regulated (Table 2). Besides, dozens of hypothetical genes (57 up-regulated, 38 down-regulated) whose function and GO category could not be determined based on their sequence homology were also found among the filament-associated genes (Table S1).

**Table 2:** Number of the filament-associated genes in the different GO categories

GO categories	Number of genes		
	Up-regulated	Down-regulated	Total number
GO:0055085 transmembrane transport	18	19	37
GO:0016192 vesicle-mediated transport	7	2	9
GO:0006913 nucleocytoplasmic transport	1	1	2
GO:0006091 generation of precursor metabolites and energy	6	7	13
GO:0006629 lipid metabolic process	8	7	15
GO:0006520 cellular amino acid metabolic process	5	9	14
GO:0016071 mRNA metabolic process	2	12	14
GO:0006399 tRNA metabolic process	1	9	10
GO:0055086 nucleobase-containing small molecule metabolic process, cofactor	6	4	10
GO:0005975 carbohydrate metabolic process	2	2	4
GO:0006766 vitamin metabolic process	1	1	2
GO:0051186 cofactor metabolic process	4	1	5
GO:0019249 lactate biosynthetic process	0	1	1
GO:0023052 signalling	6	8	14
GO:0006355 regulation of transcription, DNA-templated	11	10	21
GO:0006351 transcription, DNA-templated	1	0	1
GO:1901990 regulation of mitotic cell cycle phase transition	0	3	3
GO:0000070 mitotic sister chromatid segregation	1	1	2
GO:0140013 meiotic nuclear division	1	4	5
GO:0007163 establishment or maintenance of cell polarity	2	0	2
GO:0030036 actin cytoskeleton organization	3	1	4
GO:0000226 microtubule cytoskeleton organization	0	2	2
GO:0071554 cell wall organization or biogenesis	4	3	7
GO:0061024 membrane organization	5	3	8
GO:0042254 ribosome biogenesis	1	3	4
GO:0032200 telomere organization	2	1	3
GO:0006325 chromatin organization	4	5	9
GO:0007005 mitochondrion organization	2	5	7
GO:0140053 mitochondrial gene expression	3	6	9
GO:0005739 mitochondrion	1	2	3
GO:0005783 endoplasmic reticulum	0	1	1
GO:0005794 Golgi apparatus	0	1	1
GO:0140056 organelle localization by membrane tethering	0	1	1
GO:0098754 detoxification	2	4	6
GO:0007155 cell adhesion	1	0	1
GO:0006914 autophagy	1	1	2
GO:0006260 DNA replication	0	7	7
GO:0006281 DNA repair	0	3	1
GO:0006310 DNA recombination	1	2	3
GO:0003677 DNA binding	1	0	1
GO:0003723 RNA binding	0	1	1
GO:0002181 cytoplasmic translation	6	1	7
GO:0006457 protein folding	8	0	8
GO:0051604 protein maturation	0	4	4
GO:0030163 protein catabolic process	5	0	5
GO:0065003 protein-containing complex assembly	0	1	1
GO:0006486 protein glycosylation	0	2	2
GO:0070647 protein modification by small protein conjugation or removal	0	5	5
GO:0055065 metal ion homeostasis	1	0	1
GO:0016491 oxidoreductase activity	2	3	5
GO:0016616 oxidoreductase activity, acting on the CH-OH group of donors, NAD or NADP as acceptor	0	1	1
GO:0004145 diamine *N*-acetyltransferase activity	0	1	1
GO:0008168 methyltransferase activity	0	2	2
GO:0003959 NADPH dehydrogenase activity	1	0	0
GO:0071164 RNA trimethylguanosine synthase activity	0	1	1
GO:0036361 racemase activity, acting on amino acids and derivatives	0	1	1
GO:0008080 N-acetyltransferase activity	0	2	2

To validate our RNA sequencing results, RT-PCR analyses of intron-containing and randomly selected genes were performed (*crp79* and *meu6* were up-regulated, while the SJAG_05398 was down-regulated) (Table S1). Their RT-PCR data (Fig. 1b) were in good agreement with the RNA sequencing results. Besides, the appearance of the famous mycelial regulators *nrg1* and *fkh2* (SJAG_00124, SJAG_04813) among the up-regulated genes can also indicate reliability of the RNA sequencing data (Murad et al. 2001; Bensen et al. 2002).

### High expression of ethanol production-related genes in hyphae

**Fig. 2:**
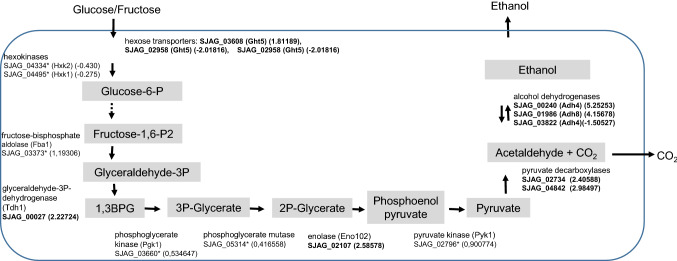
Gene expression of glycolytic and ethanol production genes in *S. japonicus* hyphae. The log_2_ fold changes are indicated in brackets. The genes whose mRNA level changed significantly in the hyphae in contrast to the yeast-phase cells, but their log_2_ value was lower than 1.5 are indicated with *

Investigating the genes and their functions, we noticed that several glycolytic genes had altered mRNA levels in the *S. japonicus* hyphae. One hexose transporter (SJAG_03608), the glyceraldehyde-3P-dehydrogenase (SJAG_00027) and enolase (SJAG_02107) were strongly up-regulated, while further glycolytic genes also showed significantly elevated mRNA levels compared to yeast cells. However, their RNA values were lower than log_2_ 1.5 (indicated with * in the Fig. 2). Interestingly, pyruvate decarboxylases (SJAG_02734, SJAG_04842) and the alcohol dehydrogenases (SJAG_00240, Adh4), (SJAG_01986, Adh8) also had increased mRNA values (Table S1, Fig. 2).

### The PKA pathway can regulate a set of the filament-associated genes

Our earlier data suggested that high cAMP level inhibited yeast-to-mycelium transition, while the *pka1* deleted cells produced shorter hyphae compared to the wild-type cells (Sipiczki et al. 1998a,b; Papp et al. 2017). Thus, we assumed that at least a portion of the mycelial genes might be regulated by the Pka1 protein. Comparison of the RNA sequencing data obtained from hypha and the *pka1* deleted strain revealed that there is an overlap between them. 75 filament-associated genes were found in the transcriptional profile of the *pka1* mutant strain (Papp et al. 2017) (Table S2). These genes could be directly or indirectly under the regulation of the PKA pathway.

### Different dimorphic yeast species share several filament-associated genes

Since mycelial growth is characteristic of different species, we wanted to learn whether there are common evolutionarily conserved filament-associated genes in these species or not. Thus, orthologous sequences of *S. japonicus* genes, whose mRNA level altered in the hyphae, were identified by reciprocal BLASTp analyses. As a result, orthologues of 49 filament-associated genes could be identified in seven dimorphic and filamentous species (*S. cerevisiae, C. albicans, Y. lipolytica, H. capsulatum, C. neoformans, U. maydis T. deformans*) (Table S3)*.* These common genes belonged to different GO categories and were often differentially regulated in the hyphae of *S. japonicus*, *C. albicans* and *H. capsulatum* (Table S3, Table 3) (Epp et al. 2010; Gilmore et al. 2015).

**Table 3:** mRNA levels of orthologous mycelial genes in *S. japonicus, C. albicans* and *H. capsulatum* hyphae compared to the yeast-phase cells

Gene identifier in *S. japonicus*	mRNA level in hyphae	Description	Gene identifier in *C. albicans*^a^	mRNA level in hyphae^a^	Gene identifier in *H. capsulatum*^b^	mRNA level in hyphae^b^
SJAG_04352	+	Cyclophilin family peptidyl-prolyl cis–trans isomerase Wis2	AOW31715 (orf19.7654)	+	HCBG_08524	+
SJAG_05015	+	NADPH dehydrogenase	orf19.3131	+	HCBG_03022	+
SJAG_04842	+	Pyruvate decarboxylase	AOW29380 (orf19.2877)	+	HCBG_06694	+
SJAG_00413	+	Acetyl-CoA C-acetyltransferase Erg10	orf19.1591	+	HCBG_07982	+
SJAG_02107	+	Enolase	AOW26488	+	HCBG_00056	+
SJAG_02734	+	Pyruvate decarboxylase	AOW29380 (orf19.2877)	+	HCBG_06694	+
SJAG_04715	–	Membrane transporter	orf19.1308	–	HCBG_06390	–
SJAG_03432	–	WDR44 family WD repeat protein	orf19.7235	–	HCBG_01676	–
SJAG_01147	–	Eukaryotic protein	orf19.6585	–	HCBG_02667	–
SJAG_00308	–	Peptide release factor	orf19.5488	–	HCBG_02802	–
SJAG_05182	–	Allantoate permease	orf19.5023	–	HCBG_02854	–
SJAG_00179	+	Glutathione S-transferase Gst2	AOW27495 (orf19.155)	+	HCBG_01438	–
SJAG_02192	+	Glucan 1,3-beta-glucosidase Bgl2	AOW28996 (orf19.4565)	+	HCBG_03629	–
SJAG_02233	+	G-protein alpha subunit	orf19.4015	+	HCBG_02983	–
SJAG_02313	+	D-3 phosphoglycerate dehydrogenase	orf19.5263	+	HCBG_02183	–
SJAG_00027	+	Glyceraldehyde-3-phosphate dehydrogenase Tdh1	orf19.6814	+	HCBG_05811	–
SJAG_03204	+	Phospholipase	orf19.6594	+	HCBG_09211	–
SJAG_02017	+	Translation elongation factor EF-1 gamma subunit	orf19.7382	+	HCBG_08684	–
SJAG_02896	+	Hydroxy-methylbilane synthase	AOW28013 (orf19.1742)	–	HCBG_01754	–
SJAG_02827	+	ER oxidoreductin Ero1a	orf19.4871	–	HCBG_01882	–
SJAG_00259	+	gar2 hipothetical protein	orf19.6090	–	HCBG_03744	–
SJAG_01768	+	Ubiquitin-specific protease	orf19.2933	–	HCBG_03115	–
SJAG_02581	+	Parasitic phase-specific protein PSP-1	orf19.24	–	HCBG_01945	–
SJAG_04268	+	HAL protein kinase Oca2	orf19.6232	–	HCBG_01745	–
SJAG_03809	+	Cdc20/Fizzy family WD repeat protein	orf19.2084	–	HCBG_03481	–
SJAG_01690	+	NADP-dependent L-serine/L-allo-threonine dehydrogenase ydfG	orf19.4633	–	HCBG_04866	+
SJAG_03794	+	DNAJ domain-containing protein Psi1	orf19.3861	–	HCBG_05481	+
SJAG_04185	+	ZIP zinc transporter Zrt1	orf19.1585	–	HCBG_07321	+
SJAG_01475	+	SAGA complex/transcription initiation factor Taf9	orf19.1111	–	HCBG_01443	+
SJAG_01725	+	Transcription factor Atf21	AOW26054	–	HCBG_06790	+
SJAG_04008	+	Cytochrome c heme lyase	orf19.4578	–	HCBG_08300	+
SJAG_03671	+	Cystathionine beta-lyase	orf19.2092	–	HCBG_07173	+
SJAG_02199	–	DNA replication ATPase	orf19.3019	–	HCBG_03457	+
SJAG_02615	–	Phenylalanyl-tRNA synthetase	orf19.2039	–	HCBG_00956	?
SJAG_01492	–	NADP-dependent L-serine/L-allo-threonine dehydrogenase yd	orf19.4633	–	HCBG_04866	+
SJAG_00307	–	Centromere-specific histone H3 CENP-A	orf19.6163	–	HCBG_02786	+
SJAG_00799	–	Ribosomal protein subunit L23	orf19.3350	–	HCBG_01756	+
SJAG_00238	–	Glutathione S-transferase Gst1	AOW27495 (orf19.155)	+	HCBG_01438	–
SJAG_04183	–	DNA replication endonuclease-helicase Dna2	orf19.1192	+	HCBG_03329	–
SJAG_02139	–	Allantoate permease//membrane transporter	orf19.5535	+	HCBG_02854	–
SJAG_02091	–	Phospholipase B Plb1	orf19.6594	+	HCBG_09211	–
SJAG_00645	–	Ubiquitin-like conjugating enzyme Atg7	orf19.707	+	HCBG_05286	–
SJAG_00674	–	Succinate-semialdehyde dehydrogenase	orf19.4543	+	HCBG_07054	–
SJAG_04085	–	Diphthamide biosynthesis protein	orf19.4173	+	HCBG_08193	+
SJAG_02958	–	Hexose transporter Ght5	orf19.2023	+	HCBG_04231	+
SJAG_03606	–	Hexose transporter Ght6	orf19.2023	+	HCBG_04231	+
SJAG_02883	–	Potassium ion transporter Trk2	orf19.600	+	HCBG_06373	+
SJAG_05329	–	Anaphase-promoting complex subunit Apc1	orf19.6046	+	HCBG_07750	+
SJAG_00493	–	Fumarylacetoacetate hydrolase	orf19.2184	+	HCBG_00180	?

+ up-regulated mRNA level, − down-regulated mRNA level compared to the wild-type yeast cells

^a^Epp et al. (2010)

^b^Gilmore et al. (2015)

### Effect of environmental factors on *S. japonicus* mycelial growth

**Fig. 3:**
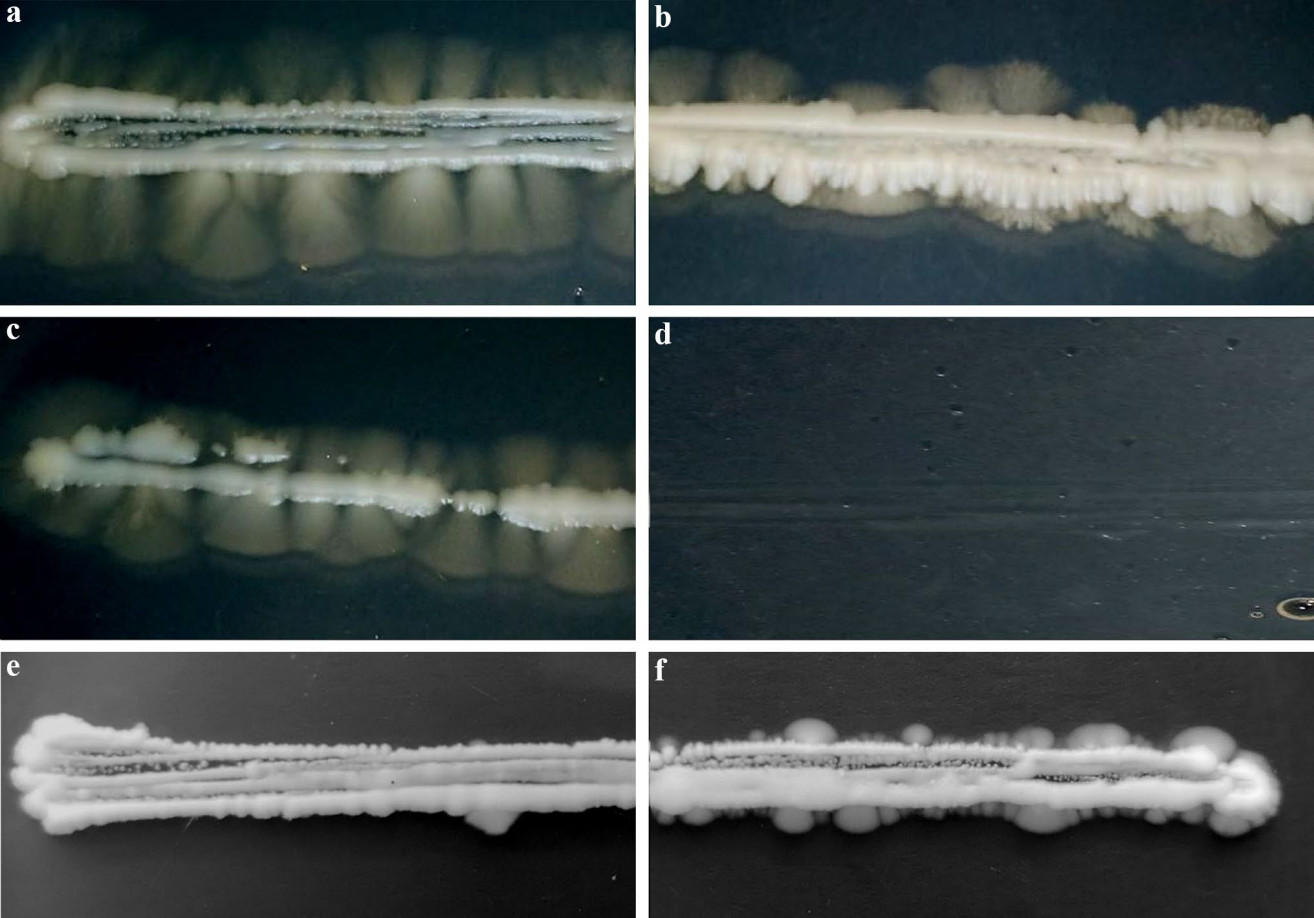
Mycelial growth of *S. japonicus* cells on different agar plates. **a** YEA (control), **b** YEA + 5% glucose, **c** YEA + 0.25% isoamyl-alcohol, (**d**) YEA + 0.5% isoamyl-alcohol. The agar plates were incubated at 30 °C for 12 days. **e** YEA and **f** YEA + 200 µM FeCl_3_ were photographed after 5 days

Since mRNA levels of several genes were different in the different dimorphic species (Table 3) (Epp et al. 2010; Gilmore et al. 2015), the question arose whether these differences could originate from the different responses to the environmental factors or not. Thus, mycelial growth and length of the *S. japonicus* hyphae were investigated on YEA culture medium (control) and YEA supplemented with isoamyl-alcohol or FeCl_3_ or higher concentration of glucose. Our result showed that higher glucose concentration (Fig. 3b) and presence of 0.25% isoamyl-alcohol (Fig. 3c) decreased length of the hyphae, compared to control plates (Fig. 3a) (average length of hyphae were 9 mm on YEA, 4 and 6 mm were on 5% glucose and 0.25% alcohol containing media). 0.5% alcohol inhibited even the cell division of the yeast-phase cells (Fig. 3d). Interestingly, the presence of 200 µM FeCl_3_ accelerated the yeast-to-hyphae transition and little hyphae appeared already after 5 days of incubation (Fig. 3f), in contrast to the YEA control plates (Fig. 3e). However, later (after 12 days) length of the hyphae was almost similar on the iron-containing and control media.

### Genome comparison between dimorphic *S. japonicus* and non-dimorphic *S. pombe* revealed some differences

**Fig. 4:**
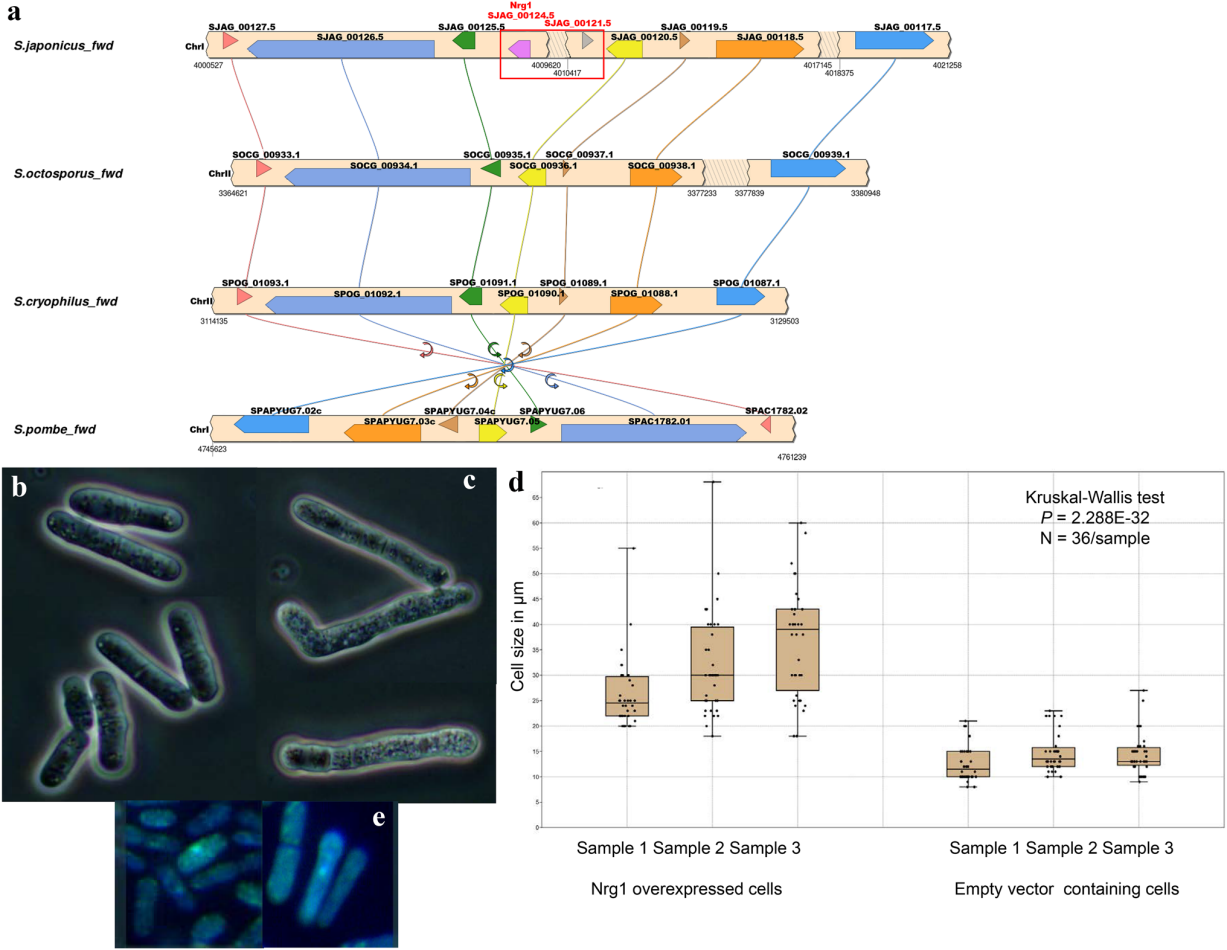
*S. japonicus nrg1* gene. *S. japonicus nrg1* gene and its neighbour SJAG_00121.5 ORF are missing from the chromosomes of the non-dimorphic *Schizosaccharomyces* species (**a**). Cell morphology of the *S. pombe* cells transformed with pREP42 empty vector (**b**) and pREP42 + *S. japonicus nrg1* gene (**c**) (EMMA, at 30 °C, after 1 day). Cell size of the transformant cells (**d**). Localisation of the Nrg1-GFP protein (**e**)

Since we failed to identify the homologues of several *S. japonicus* filament-associated genes in the non-dimorphic but closely related *S. pombe* sequence by reciprocal BLASTp analyses, we wanted to know whether *S. pombe* homologous genes were missing or not. Since gene content and -structure seem to be remarkably conserved in the fission yeast clade, a synteny analysis was performed between the *S. pombe* and *S. japonicus* genomes (Rhind et al. 2011; Ács-Szabó et al. 2018). This analysis confirmed the lack of several genes in the non-dimorphic *S. pombe* genome (Table S4), including the known regulator of hyphae production, the *nrg1* (Gómez-Gil et al. 2019; Braun et al. 2001; Murad et al. 2001; Kuchin et al. 2002) (Table S4). The synteny analysis clearly showed that the chromosome fragment, which contains the *nrg1* (SJAG_00124) gene and the neighbour SJAG_00121.5 ORF, are missing from the non-dimorphic *S. pombe* chromosome*,* while their 5′ and 3′ adjacent genes are present in it (Fig. 4a) (Table S5). Further analyses showed that this chromosome fragment is also missing from the other related and non-dimorphic fission yeast species, *S. octosporus* and *S. cryophilus* (Fig. 4a) (Table S5).

**Fig. 5:**
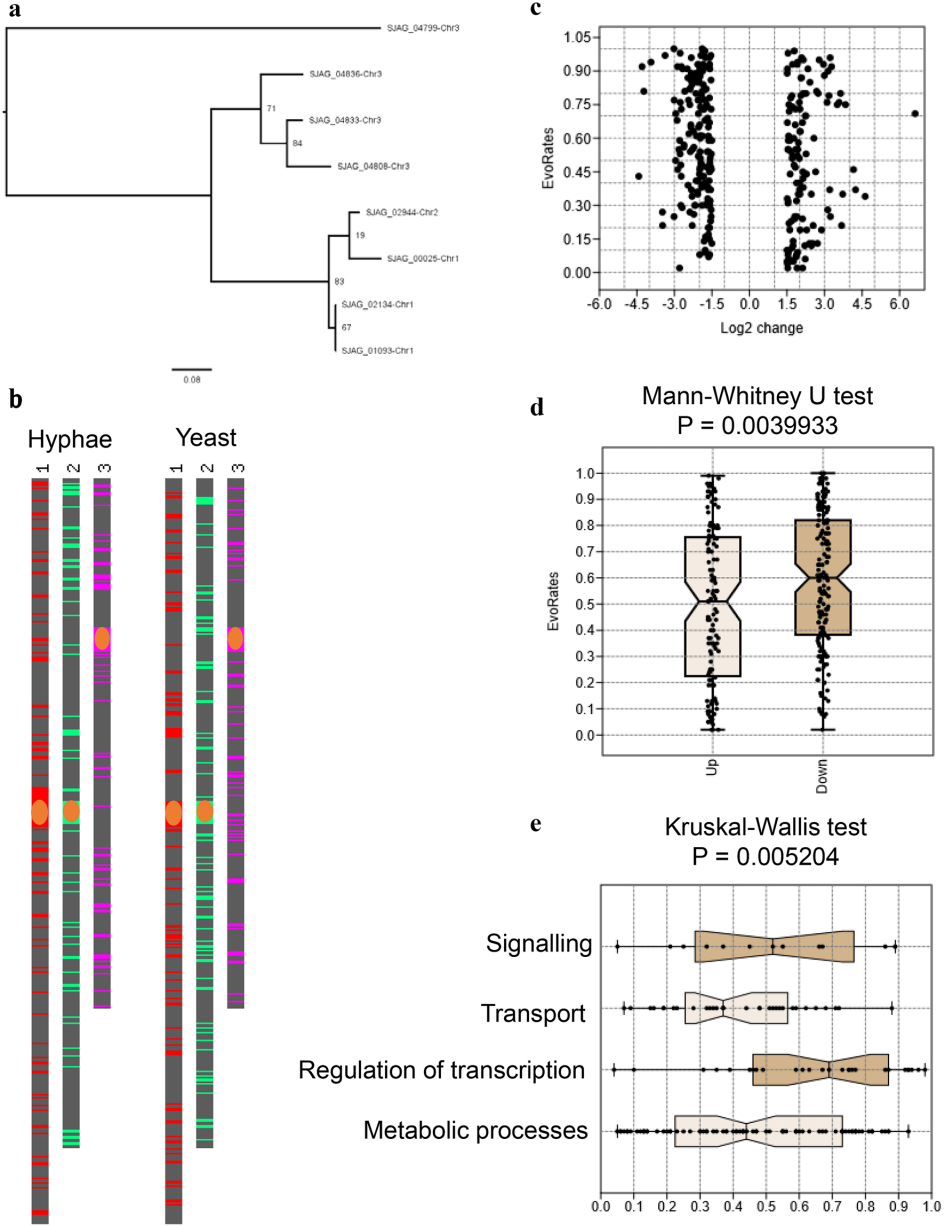
Bioinformatic analyses of filament-associated genes. Phylogenetic analysis of paralogous genes showed that the genes localised on the same chromosome had higher sequence similarity (**a**). Filament-associated genes localised equally to all three *S. japonicus* chromosomes (**b**). (Orange ellipses represent centromers) Evolutionary rates of differentially expressed genes were varied (**c**). Higher evolutionary rates were found in the case of the down-regulated genes compared to up-regulated mycelial genes (**d**). Transcriptional regulators had significantly higher evolutionary rates than those genes which were involved in transport and metabolic processes (**e**). (Dunn’s post hoc test with Bonferroni correction, *P* = 0.0051 and *P* = 0.01203, respectively)

Besides the missing genes, we found also a multicopy gene (SJAG_04836) among the filament-associated genes, too, which had several paralogs in the *S. japonicus* genome. Interestingly, all paralogs showed elevated mRNA levels in the hyphae, compared to the yeast cells (Table 4). These paralogous genes were located on all three chromosomes (Table 4) (Fig. 5a), similarly to other mycelial genes (Fig. 5b). However, their filogenetic analysis showed that genes localised on the same chromosome had higher sequence similarity (Fig. 5a).

**Table 4:** Paralogous mycelial genes in the *S. japonicus* genome

Paralogs in the *S. japonicus genome*	Log_2_ (fold_change) in hyphae	Significant	*S. pombe* homologues	Localisation on the *S. japonicus* chromosomes
SJAG_04836 hypothetical protein	5.92286	Yes	No	3
SJAG_01093 hypothetical protein	4.59153	Yes	No	1
SJAG_04808 hypothetical protein	3.76076	Yes	No	3
SJAG_00025 hypothetical protein	3.23770	Yes	No	1
SJAG_02944 hypothetical protein	2.17468	Yes	No	2
SJAG_04833 hypothetical protein	1.72023	Yes	No	3
SJAG_02134 hypothetical protein	1.37	Yes	No	1
SJAG_04799 hypothetical protein	0.66	Yes	No	3^a^

^a^Reverse orientation https://fungi.ensembl.org/

### Transformation of the *S. japonicus nrg1* gene into the *S. pombe* cells

Since mRNA level of the *nrg1* was high in the *S. japonicus* hyphae (Table S4) and at the same time the *nrg1* homologous gene was missing from the *S. pombe* genome (Tables S4, S5), we assumed that its absence could have greatly contributed to the fact that *S. pombe* cells are not able to produce true hyphae. To test this assumption, the *S. japonicus nrg1* gene was cloned into an *S. pombe*-specific vector (pREP42-GFP) and this DNA construction was transformed into a uracil auxotrophic *S. pombe* strain (2-1210). Morphology of the transformant cells was investigated on EMMA minimal medium, where the nmt promoter was induced (30 °C). However, the transformed cells were not able to form true hyphae, the *S. pombe* cells having the *nrg1-*containing vector showed frequently longer cell shape (Fig. 4c) compared to the control cells (Fig. 4b). Ratio of the longer cells was 47% in the cell population having the pREP42 + *nrg1* vector, and 8% in the empty vector containing cells. The average size was 12–13 µm in the control cells and more than 20 µm in the *nrg1* over-expressed cells (Fig. 4d). The Nrg1 protein was localised to the nucleus (Fig. 4e).

### Evolutionary rate of mycelial genes

We assumed that not only the presence of certain hyphae-specific genes, but their greater flexibility could also have contributed to the greater morphological complexity of *S. japonicus*. To prove this assumption, evolutionary rate of the filament-associated genes was collected and analysed (Rhind et al. 2011). The analyses suggested that although a wide range of evolutionary rates is typical of mycelial genes (Fig. 5c), a little bit higher evolutionary rate was found in the case of down-regulated genes (Fig. 5d). Interestingly, further analyses also revealed that regulators, especially transcriptional regulators could have significantly higher evolutionary rate than those genes which are involved in the transport and metabolic processes (Fig. 5e).

## Discussion

Dimorphic *S. japonicus* belongs to a divergent and early separated branch of the Ascomycete fungi, which is only distantly related to the budding yeasts (Sipiczki 2000; Kuramae et al. 2006). In addition, this clade contains three additional non-dimorphic species (*S. pombe, S. octosporus, S. cryophylus*), which have remarkably conserved gene- content and structure (Rhind et al. 2011; Ács-Szabó et al. 2018). Taking advantage of these opportunities, our aim was to reveal similarities and differences of mycelial growth of the different yeast species and find deviations, if there are, in the genomes of the dimorphic and non-dimorphic fission yeasts.

To obtain insight into the mycelial genes of *S. japonicus*, RNA sequencing was performed in wild-type hyphae and yeast-phase cells. 403 genes (which had at least ± 1.5 or higher average log_2_ value) were differentially expressed in hyphae compared to yeast-phase cells. These genes had varied functions and belonged to very different GO categories, similar to the *Candida* mycelial genes (Nantel et al. 2002; Kadosh and Johnson 2005; Carlisle and Kadosh 2013; Wu et al. 2016), suggesting that production of hyphae can be a quite complex process. Although *Candida* and *S. japonicus* are distantly related species, comparison of their transcriptional profiling data showed that the transport- and metabolic genes were involved in high number in their hyphal growth (Kadosh and Johnson 2005; Wu et al. 2016)*.* A further similarity is that the *nrg1* and *fkh2* genes can be important mycelial regulators in both species (Murad et al. 2001; Bensen et al. 2002).

We also noticed that the pyruvate decarboxylases (SJAG_02734, SJAG_04842) or alcohol dehydrogenases (SJAG_00240, Adh4), (SJAG_01986, Adh8) had elevated mRNA levels in the *S. japonicus* hyphae. These data suggest that strong glycolysis, ethanol and CO_2_ production may characterise mycelial growth. This result can be in good agreement with the phenotype of the *C. albicans* TYE7∆ strain, which suggests a relationship between expression of glycolytic genes and biofilm formation (Bonhomme et al. 2011). The strong fermentative processes of the hyphae can be in connection with the fact that *S. japonicus* frequently produce true invasive hyphae, which penetrate the medium, where there are oxygen-poor conditions (Sipiczki et al. 1998a).

Comparison of our data with further distantly related dimorphic species (*S. cerevisiae, C. albicans, Y. lipolytica, H. capsulatum, C. neoformans, U. maydis T. deformans*) revealed that these species shared 49 common filament-associated genes. Regulation of these evolutionarily conserved genes can partly be different in *S*. *japonicus, C. albicans* and *H. capsulatum* mycelia (Epp et al. 2010 and Gilmore et al. 2015), because their mRNA levels ran sometimes in opposite directions. However, these differences can also originate from the different responses to environmental factors. Since the environmental and stress factors have a strong impact on hyphae production (reviewed in Biswas et al. 2007), we tested the effect of some culture factors on the *S. japonicus* mycelial growth. The presence of isoamyl-alcohol did not induce hyphal development in *S. japonicus,* unlike budding yeasts, instead it slightly decreased length of the hyphae (Ceccato-Antonini and da Silva 2002; Dickinson 1996). We also obtained different results from budding yeasts when we applied FeCl_3_ supplementation in the medium. Iron supplementation prevented the mycelial growth of *Candida* (Hameed et al. 2008), but increased the yeast-to-hyphae transition in the *S. japonicus* cells, which, however, produced a similar length of hyphae after longer incubation time as control cells. In contrast, the lower glucose concentration favoured mycelial growth, similarly to the budding yeasts (Cullen and Sprague 2000; Buu and Chen 2014). These findings were consistent with the previous results, which showed that *S. japonicus* cells responded to environmental changes partly similarly (FBS induction), partly differently (pH), compared to the *Candida* species (Mackenzie 1962; Joshi et al. 1973; Perez-Campo and Dominguez 2001; Kim et al. 2000; Papp et al. 2014; Konno et al. 2006).

Further comparisons confirmed the role of the PKA pathway in the morphological transition. Comparison of the transcriptional profiling data obtained from *S. japonicus* hyphae and *pka1* deleted cells showed that there was an overlap between them (Papp et al. 2017). This is in good agreement with our previous observations that cAMP level and the *pka1* mutation can influence *S*. *japonicus* hyphae production (Sipiczki et al. 1998a,b; Papp et al. 2017), and that PKA pathway is involved in the filamentous growth of several yeast species (reviewed in Biswas et al. 2007; Giacometti et al. 2011; Pan and Heitman 1999).

Our further studies focused on the differences between the genomes of *S. japonicus* and its non-dimorphic cousin, *S. pombe*. Sequence alignments and synteny analyses were carried out and they showed that one group of filament–associated genes (almost one hundred genes) was missing from the *S. pombe* genome. Unexpectedly, the known regulator of mycelial growth, the *nrg1* (Murad et al. 2001; Braun et al. 2001) and its adjacent ORF (SJAG_00121.5) were also among the missing genes, while their 5’ and 3’ neighbour genes could be found in the *S. pombe* chromosome. The synteny analyses also pointed to the fact that these two genes have been eliminated from the chromosome early, because they were also missing from the genomes of their other non-dimorphic cousins, *S. cryophylus*, *S. octosporus*.

As for the role of *nrg1* gene, our RNA sequencing data seems to confirm that it can have a strong role as an activator in the *S. japonicus* filamentous growth (its log_2_ value was 4.05163 in the hyphae), as it was suggested by the previous results of Gomez-Gil et al. (2019), in contrast to *Candida NRG1*, which has a repressor effect (Braun et al. 2001; Murad et al. 2001). However, this gene alone was not able to induce filamentous growth in non-dimorphic *S. pombe* cells when we cloned and transformed it into this closely related species. We assume that other missing mycelial genes could also be necessary for dimorphic capacity. This assumption is supported by the fact that we found, for example, a japonicus-specific gene (SJAG_04836) which had seven paralogs in the *S. japonicus* genome and all of them showed elevated mRNA levels in the hyphae. Expansion of certain genes can be in good agreement with the findings that genomes of filamentous fungi can contain more genes than non-dimorphic species (Soanes et al. 2008). However, fission yeast species have quite similar gene content (Rhind et al. 2011). Besides the chromosomal mutations, the higher evolutionary rate of the down-regulated mycelial genes and the regulator genes could also contribute to the higher morphological capacity of *S. japonicus*.

Taken together, this study revealed the filament-associated genes of the wild-type *S. japonicus* strain. We identified the common mycelial genes of different dimorphic yeast species and shed light on some similar features of the hyphae production of budding and fission yeasts. We confirmed the regulatory role of the PKA pathway in the hyphal growth and pointed to the fact that chromosomal changes could have contributed to loss of filamentous growth in non-dimorphic species and preservation of *S. japonicus* dimorphic capacity. We believe that all these results can provide valuable information about the dimorphic capacity of yeasts and the genomic background behind it.

## Supplementary Information

Below is the link to the electronic supplementary material.

**Table S1 **The *S. japonicus *mycelial genes, their functions and GO categories (XLSX 59 KB)

**Table S2 **The PKA pathway regulated mycelial genes (DOCX 21 KB)

**Table S3 **The conserved filament-associated genes and their orthologues in the different dimorphic yeast species (XLSX 21 KB)

**Table S4 **List of the filament-associated genes which were missing in *S. pombe* genome based on Blastp and synteny analyses (XLSX 1073 KB)

**Table S5 **The *nrg1* gene and its adjacent ORF are missing from genomes of the non-dimorphic fission yeasts (*S. pombe, S. octosporus, S. cryophylus)* based on Blastp and synteny analyses (XLSX 11 KB)

## Data Availability

All data generated or analysed during this study are included in this published article and its supplementary information files.
