# Supplementary material for: Molecular and comparative genomic analyses reveal evolutionarily conserved and unique features of the Schizosaccharomyces japonicus mycelial growth and the underlying genomic changes
Source: Curr Genet. 2021 Aug 24;67(6):953–68. doi: 10.1007/s00294-021-01206-y (PMC8594269; doi:10.1007/s00294-021-01206-y)
Supplement: Supplementary file 2 — Table S2 The PKA pathway regulated mycelial genes (DOCX 21 KB) [file 294_2021_1206_MOESM2_ESM.docx]

| **Genes and their functions** | **changes of the mRNA level in hyphae** | **changes of the mRNA level in the**  ***pka1* mutant strain*** |
| --- | --- | --- |
| SJAG_00027 glyceraldehyde-3-phosphate dehydrogenase Tdh1 | + | + |
| SJAG_02233 G-protein alpha subunit | + | + |
| SJAG_02968 iron/zinc ion transporter | + | + |
| SJAG_01096 amino acid permease | + | + |
| SJAG_02191 GFO/IDH/MocA family oxidoreductase | + | + |
| SJAG_02734 pyruvate decarboxylase | + | + |
| SJAG_01553 hypothetical protein | + | + |
| SJAG_03204 phospholipase | + | + |
| SJAG_01416 pig-L | + | + |
| SJAG_00110 But2 family protein | + | + |
| SJAG_01846 But2 family protein But2 | + | + |
| SJAG_03653 thioredoxin reductase Trr1 | + | + |
| SJAG_02788 fungal protein | + | + |
| SJAG_01477 fungal protein | + | + |
| SJAG_01095 hypothetical protein | + | + |
| SJAG_02784 hypothetical protein | + | + |
| SJAG_03390 hypothetical protein | + | + |
| SJAG_03815 hsp16-like protein //hypothetical protein | + | + |
| SJAG_03203 hypothetical protein | + | + |
| SJAG_02883 potassium ion transporter Trk2 | - | - |
| SJAG_04939 bis(5'-adenosyl)-triphosphatase | - | - |
| SJAG_04085 diphthamide biosynthesis protein | - | - |
| SJAG_00308 peptide release factor | - | - |
| SJAG_01607 hypothetical protein | - | - |
| SJAG_06631 hypothetical protein | - | - |
| SJAG_01525 hypothetical protein | - | - |
| SJAG_05745 hypothetical protein | - | - |
| SJAG_02708 RecA family ATPase Rhp55 | - | - |
| SJAG_04636 hypothetical protein | - | - |
| SJAG_00238 glutathione S-transferase Gst1 | - | - |
| SJAG_00277 hypothetical protein | - | - |
| SJAG_00165 hypothetical protein | - | - |
| SJAG_05250 hypothetical protein | + | - |
| SJAG_03600 rho guanine nucleotide exchange factor | + | - |
| SJAG_04813 fork head transcription factor Fkh2 | + | - |
| SJAG_02113 amino acid permease | + | - |
| SJAG_01836 CAF1 family ribonuclease | + | - |
| SJAG_02124 cobW | + | - |
| SJAG_04696 kinetochore protein Mis15 | + | - |
| SJAG_04575 meiotic chromosome segregation protein Meu6 | + | - |
| SJAG_04303 meiotic recombination protein Rec25 | + | - |
| SJAG_00100 meiosis specific cyclin Crs1 | + | - |
| SJAG_02710 hypothetical protein | + | - |
| SJAG_00703 hypothetical protein//DUF1674 family protein | + | - |
| SJAG_00354 hypothetical protein | + | - |
| SJAG_00025 hypothetical protein | + | - |
| SJAG_00018 hypothetical protein | + | - |
| SJAG_02944 hypothetical protein | + | - |
| SJAG_04833 hypothetical protein | + | - |
| SJAG_01421 hypothetical protein//sequence orphan | + | - |
| SJAG_01450 hypothetical protein | + | - |
| SJAG_02940 tr 3-O-acetyltransferase | + | - |
| SJAG_01690 NADP-dependent L-serine/L-allo-threonine dehydrogenase ydfG | + | - |
| SJAG_03822 alcohol dehydrogenase Adh4 | - | + |
| SJAG_02148 glucose-6-phosphate 1-dehydrogenase | - | + |
| SJAG_02951 UDP-glucose 4-epimerase/aldose 1-epimerase, gal10 | - | + |
| SJAG_00699 tspO/peripheral benzodiazepine receptor | - | + |
| SJAG_05182 allantoate permease | - | + |
| SJAG_04299 uricase | - | + |
| SJAG_03825 UbiE family methyltransferase | - | + |
| SJAG_00781 P-factor pheromone Map2 | - | + |
| SJAG_00223 hsp9-like protein | - | + |
| SJAG_00674 succinate-semialdehyde dehydrogenase | - | + |
| SJAG_04376 peptidase | - | + |
| SJAG_02886 gamma tubulin complex subunit Mod21 | - | + |
| SJAG_03526 Lsd90 protein | - | + |
| SJAG_02091 phospholipase B Plb1 | - | + |
| SJAG_02341 ubiquitin carboxy terminal hydrolase Uch2 | - | + |
| SJAG_03303 manganese superoxide dismutase | - | + |
| SJAG_01965 hypothetical protein | - | + |
| SJAG_04674 hypothetical protein | - | + |
| SJAG_02960 flavonol reductase/cinnamoyl-CoA reductase family protein | - | + |
| SJAG_01578 fungal protein | - | + |
| SJAG_05005 fungal protein | - | + |
| SJAG_02442 hypothetical protein | - | + |

*Papp et al. 2017

+ up-regulated, - down-regulated mRNA level compared to the wild-type yeast cells
